# Influencing factors of knowledge proficiency of general practitioners in rural China for esophageal cancer prevention and treatment: a cross-sectional study

**DOI:** 10.1017/S1463423623000701

**Published:** 2024-02-12

**Authors:** Jinjia Zhang, Huadong Wu, Rongying Wang, Min Zhang

**Affiliations:** 1 Department of General Practice, The Second Hospital of Hebei Medical University, Shijiazhuang Hebei, China; 2 Department of Gastrointestinal Surgery, The Second Hospital of Hebei Medical University, Shijiazhuang, Hebei, China

**Keywords:** esophageal cancer, general practitioners, knowledge proficiency

## Abstract

**Background:**

This study aims to investigate the knowledge of rural general practitioners (GPs) in esophageal cancer (EC) prevention and treatment in China and analyze relevant influencing factors, so as to improve the ability of rural GPs in EC prevention and treatment.

**Methods:**

This cross-sectional study was conducted from November 5, 2021, to November 20, 2021. A self-designed questionnaire was used to conduct an online survey. Multivariable logistic regression models were used to identify the influencing factors of knowledge proficiency of GPs in rural China for EC prevention and treatment.

**Results:**

This study included 348 participants from 12 rural areas in Hebei Province. The mean accuracy rate on all question items was 42.3% ± 10.67%. Sex (OR = 2.870, 95% CI: 1.519–5.423), educational level (OR = 3.256, 95% CI: 1.135–9.339), and comprehension of clinical practice guidelines for EC (OR = 4.305, 95% CI: 2.023–9.161) were significant predictors for GPs’ knowledge proficiency of EC prevention and treatment (*P* < 0.05).

**Conclusions:**

The study indicated that knowledge proficiency of rural GPs of EC prevention and control still awaits to be improved. Sex, educational level, and comprehension of clinical practice guidelines for EC were significant predictors for their proficiency.

## Introduction

Esophageal cancer (EC) refers to malignant carcinoma originating from esophageal mucosal epithelium, which is one of the most commonly seen fatal malignant tumors all over the world. According to the worldwide cancer statistics, the incidence and mortality of EC ranked seventh and sixth, respectively, in the year of 2018 (Bray *et al.*, 2018). The incidence of EC in China is significantly higher than those in other countries. In 2018, the incidence rate of EC in China was 13.9/100,000, which was approximately 2.21 times of the global statistics at the same time. In addition, domestic cancer statistics also suggested a mortality rate of 12.7/100,000, which was about 2.31 times of the global cancer deaths caused by EC, resulting in EC as the fourth leading cause for cancer death among all carcinoma in China (He *et al.*, 2017; Bray *et al.*, 2018). Besides, distribution of EC histologic subtypes presents obvious geographical differences. At the national level, the most common domestic histologic subtype is squamous cell carcinoma, with about 50.3% of the global squamous cell carcinoma cases distributed in China (Lin *et al.*, 2022). More importantly, statistics also indicated a trend of inequality of epidemiological features of EC between Chinese rural and urban areas, with the incidence in rural areas 2 to 10 times higher than that in urban areas (Yuan and Xie, 2021), which is speculated to be associated with higher prevalence of major risk factors for EC in rural areas, including lower socioeconomic status, smoking, heavy drinking, and dietary factors (Zhou *et al.*, 2006; Dong and Thrift, 2017).

The prognosis of EC largely depends on the clinical stage at the time of diagnosis. More than 90% of the patients with EC at early stage may survive for at least 5 years after receiving minimally invasive treatment, while the 5-year survival rate among patients with advanced EC is still less than 30% following multiple anticancer treatments, such as surgery, radiotherapy, and chemotherapy (Min *et al.*, 2018; Zeng *et al.*, 2018). However, due to the stealthiness of tumor at early stage, more than 90% of the EC patients would not get diagnosed until advanced stage (DiSiena *et al.*, 2021). From the perspective of EC disease progression, the precancerous state of EC may last for 5–10 years, and the prognosis shall be significantly improved if effective preventions were applied during the window. Prevention strategies are divided into primary prevention and secondary prevention according to different focuses. The former one mainly focus on proactive control of risk factors for EC by changing unhealthy lifestyles, while the later one relies on early diagnosis and treatment, which can be achieved through EC screening. Therefore, screening programs among people at high risks for EC are of vital importance to, thereby facilitating the introduction of interventions at early stage. Currently, electronic gastroscopy is the most commonly used method for EC screening (Hosseini *et al.*, 2023), which is less used in rural areas due to lack of gastroscopy equipment, qualified technicians, and healthcare consciousness (Wu *et al.*, 2017; Jia *et al.*, 2019), all of which may hinder the widespread application of gastroscopy screening in China for potential EC.

Doctors’ knowledge proficiency toward EC can determine their prescribing process. However, current studies on EC mainly focus on blood chemistry markers or genes, and there are few studies on GPs’ knowledge proficiency of EC (Ji Q *et al.*, 2016; Picard M, 2022). Cross-sectional research has its advantages in understanding knowledge, attitudes, and practices. Therefore, this cross-sectional study aims to investigate the knowledge of rural general practitioners (GPs) in EC prevention and treatment in China and analyze relevant influencing factors, so as to improve the ability of rural GPs in EC prevention and treatment.

## Methods

The survey was designed in accordance with ethical principles indicated in Declaration of Helsinki. Anonymous questionnaire was adopted for data collection. The introduction page of the questionnaire contained information about informed consent, and participants provided their consent by clicking the “Start” button. The protocol of this study was approved by the Ethics Committee of the Second Hospital of Hebei Medical University (No. 2021-R321).

### Research participants

Participants from the rural district in Fuping Xian of Baoding, China, were enrolled to this cross-sectional study via cluster sampling, and data from November 5, 2021, to November 20, 2021, were collected for analyses. Due to the severe shortage of GPs in rural areas, village doctors were included. The total number of GPs in rural Fuping is around 400. In total, 366 GPs agreed to participate. Finally, excluding 18 incomplete questionnaires, 348 valid questionnaires were completed with 95.08% (348/366) effective rate.

### Research methods

The questionnaire used in this study was designed after consulting National Comprehensive Cancer Network Guidelines (Ajani *et al.*, 2015), European Society for Medical Oncology Clinical Practice Guidelines (Stahl *et al.*, 2013), and large prospective cohort studies conducted in China (Chen *et al.*, 2019) and South Korea (Cho *et al.*, 2020). Then the expert group revised the initial draft. Afterward, we conducted a presurvey and modified the items in the questionnaire again to form the final questionnaire (Table S1).The final questionnaire was filled out on the Internet platform (star network platform) powered by www.wjx.cn. Participant recruiting was carried out by publishing survey advertisements in the WeChat group, which is the most widely used social networking software in China. The same internet protocol address could only be filled out once. The questionnaire consisted of two major domains: GPs’ general status and GPs’ knowledge in EC prevention and treatment. The former domain included age, sex, educational level, professional title, specialty, years of work experience, working hours per week, number of clinic patients per day, and income per month. The later domain included 20 items covering four aspects: risk factor identification, screening and diagnosis, treatment methods for different stages, disease surveillance and follow-up.

EC prevention and treatment knowledge mastery was classified based on the number of correct answers. Participants with above-average scores were assigned to the group with in-depth knowledge in EC prevention and treatment, while those with below-average scores were assigned to the other group (Li Y *et al.*, 2021).

### Statistical analysis

Statistical analysis was performed using SPSS 20.0 statistical software. Enumeration data were expressed in the formation of relative numbers. The *χ* ^2^ test was employed for intergroup comparison, while the Wilcoxon rank-sum test was adopted for rank data comparison. Multivariate analysis was conducted through logistic regression analysis, the result of which was employed to establish the risk prediction model and obtain joint predictors. The risk prediction model was verified using a series of parameters of the joint predictor, including sensitivity, specificity, Youden index (YI), positive and negative predictive values, receiver operator curves (ROC), and area under curve (AUC). A *P* value less than 0.05 was considered statistically significant.

## Results

This study included 348 participants from 12 rural areas in Hebei Province. Among all participants included in the study, 277 (73.0%) participants were male, and 71 (27.0%) participants were female. The median age was 55.8 years (range: 28–76 years). The most advanced degree of 155 (44.5%) participants were senior high school or below, 172 (49.4%) participants were associate’s degree, and only 21 (6.0%) participants obtained bachelor’s degree or above. Other demographic characteristics such as professional title, specialty, and years of work experience are summarized in Table 1.

**Table 1.** General status of general practitioners

		Total (*n *= 348)	Esophageal cancer prevention and treatment knowledge mastery		*χ* ^2^	*P*
			Low knowledge score group (*n* = 196)	High knowledge score group (*n* = 152)		
Age (year)	<35	10 (2.9%)	3 (30.0%)	7 (70.0%)	8.565	**0.036**
	35–44	81 (23.3%)	37 (45.7%)	44 (54.3%)		
	45–54	98 (28.2%)	59 (60.2%)	39 (39.8%)		
	≥55	159 (45.6%)	97 (61.0%)	62 (39.0%)		
Sex	Male	277 (79.6%)	176 (63.5%)	101 (36.5%)	28.738	**<0.001**
	Female	71 (20.4%)	20 (28.2%)	51 (71.8%)		
Educational level	Senior high school	155 (44.5%)	89 (57.4%)	66 (42.6%)	7.052	**0.029**
	Associate’s degree	172 (49.4%)	101 (58.7%)	71 (41.3%)		
	Bachelor’s degree or above	21 (6.1%)	6 (28.6%)	15 (71.4%)		
Professional title	Beginner	99 (28.4%)	61 (61.6%)	38 (38.4%)	4.005	0.135
	Intermediate	244 (70.1%)	134 (54.9%)	110 (45.1%)		
	Vice-senior and above	5 (1.4%)	1 (20.0%)	4 (80.0%)		
Specialty	Clinical medicine	293 (84.2%)	167 (57.0%)	126 (43.0%)	0.492	0.782
	Traditional Chinese medicine practitioner	41 (11.8%)	21 (51.2%)	20 (48.8%)		
	Others	14 (4.0%)	8 (57.1%)	6 (42.9%)		
Years of work experience	<5 years	20 (5.7%)	10 (50.0%)	10 (50.0%)	3.921	0.141
	5–9 years	78 (22.4%)	37 (47.4%)	41 (52.6%)		
	≥10 years	250 (71.9%)	149 (59.6%)	101 (40.4%)		
Working hours per week(h)	<40	151 (43.4%)	90 (59.6%)	61 (40.4%)	1.865	0.393
	40–55	123(35.3%)	69 (56.1%)	54 (43.9%)		
	≥56	74(21.3%)	37 (50.0%)	37 (50.0%)		
Clinic patients per day	<10	153 (44.0%)	83 (54.2%)	70 (45.8%)	2.340	0.310
	10–19	118 (33.9%)	73 (61.9%)	45 (38.1%)		
	≥20	77 (22.1%)	40 (51.9%)	37 (48.1%)		
Income per month (CNY)	<1000	41 (11.8%)	27 (65.9%)	14 (34.1%)	4.138	0.126
	1000–2999	231 (66.4%)	133 (57.6%)	98 (42.4%)		
	≥3000	76 (21.8%)	36 (47.4%)	40 (52.6%)		
Comprehension of clinical practice guidelines for EC	No	294 (84.5%)	185 (62.9%)	109 (37.1%)	33.583	**<0.001**
	Yes	54 (15.5%)	11 (20.4%)	43 (79.6%)		

Regarding the domain of GPs’ knowledge in EC prevention and treatment, the mean accuracy rate on all question items was 42.3% ± 10.67%, and the average accuracy rates of the four sub-aspects (risk factor identification, screening and diagnosis, treatment method for different stages, disease surveillance, and follow-up) were 37.8% ± 12.35%, 41.5% ± 9.48%, 67.2% ± 13.78%, and 31.8% ± 10.25%, respectively.

**Figure 1.**
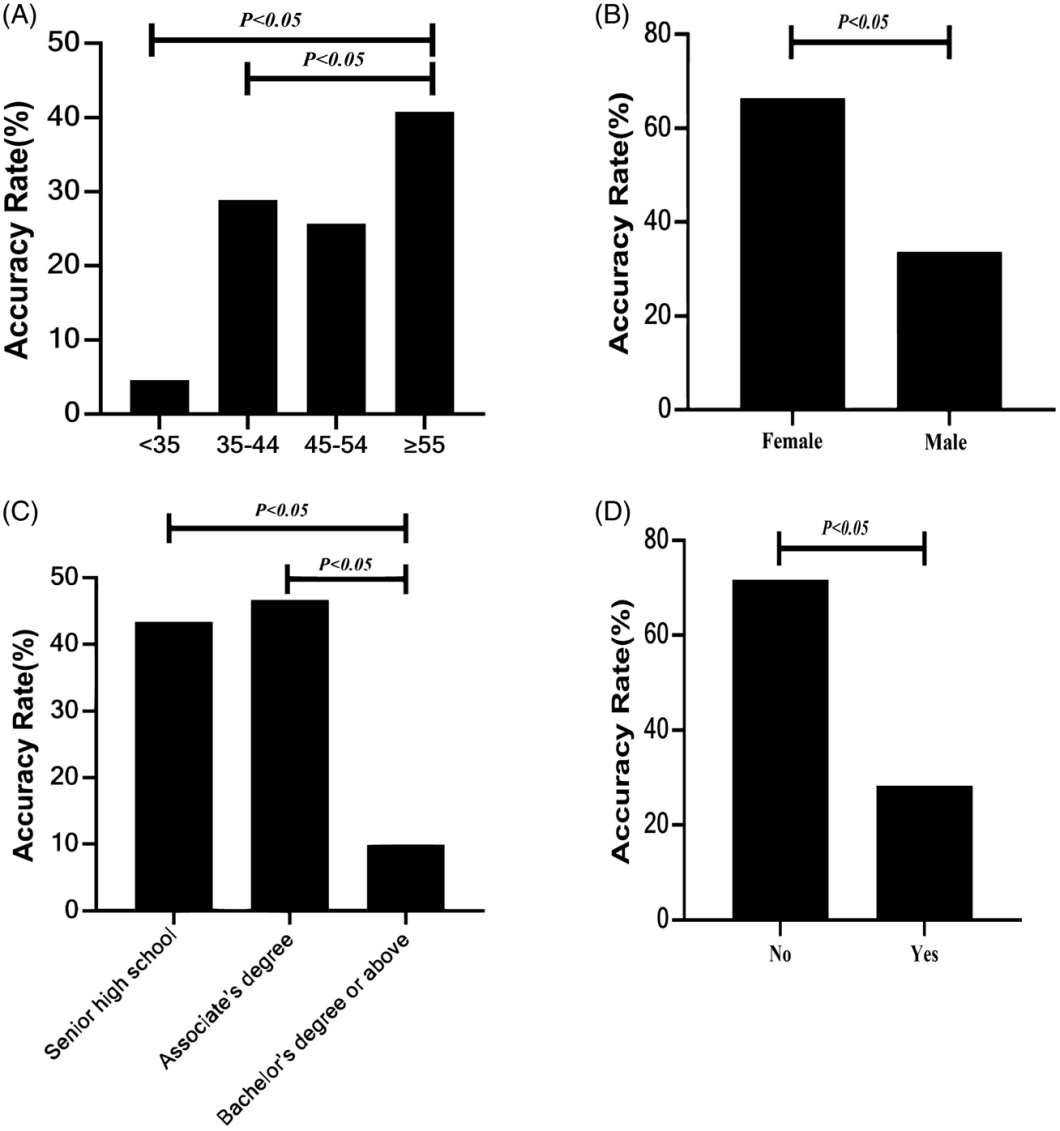
Comparison of knowledge proficiency of GPs for EC prevention and treatment in different groups (A: age; B: sex; C: educational level; D: comprehension of clinical practice guidelines for EC). Legends: Compared with GPs in the low knowledge score group, those with high scores were significantly older (Fig. 1A) and had more advanced degrees (Fig. 1C). Sex (Fig. 1B) and comprehension of clinical practice guidelines for EC (Fig. 1D) also affected their of knowledge proficiency of EC prevention and treatment.

On average, the number of questions with correct answer was 8.12 ± 4.56 for each GP. Therefore, a score of 8 was set as the threshold, and the GPs were divided into two groups accordingly, that GPs with scores lower than 8 were assigned to the low knowledge score group and the others were assigned to the high knowledge score group. Differences between the two groups are shown in Table 1. Compared with GPs in the low knowledge score group, those with high scores were significantly older (Fig. 1A) and had more advanced degrees (Fig. 1C). In addition, sex (Fig. 1B) and comprehension of clinical practice guidelines for EC (Fig. 1D) also affected their of knowledge proficiency of EC prevention and treatment.

In the multivariate analysis, binary logistic regression analysis was conducted with the EC prevention and treatment knowledge scores as the dependent variable (assignment: low knowledge score group = 0; high knowledge score group = 1). The results showed that sex (OR = 2.870, 95% CI: 1.519–5.423), educational level (OR = 3.256, 95% CI: 1.135–9.339), and comprehension of clinical practice guidelines for EC (OR = 4.305, 95% CI: 2.023–9.161) were significant predictors for GPs’ knowledge proficiency of EC prevention and treatment (*P* < 0.05, Table 2).

**Table 2.** Result of multivariate logistic regression analysis

Independent variable	***B***	SE	Wald *χ* ^2^ value	*P* value	OR (95% CI)
Sex (male *vs* female)	1.054	0.325	10.552	0.001	2.870 (1.519–5.423)
Educational level (senior high school or below *vs* bachelor’s degree or above)	1.181	0.538	4.823	0.028	3.256 (1.135–9.339)
Comprehension of clinical practice guidelines for EC (no *vs* yes)	1.460	0.385	14.357	<0.001	4.305 (2.023–9.161)

Based on the results of logistic regression analysis, the joint predictor (*L*) was obtained using the following formula: *L* = *X*_1_ + 1.120 *X*_2_ + 1.385*X*_3_, where *X*_1_ denotes sex, *X*_2_ denotes educational level, and *X*_3_ denotes comprehension of clinical practice guidelines for EC.

**Figure 2.**
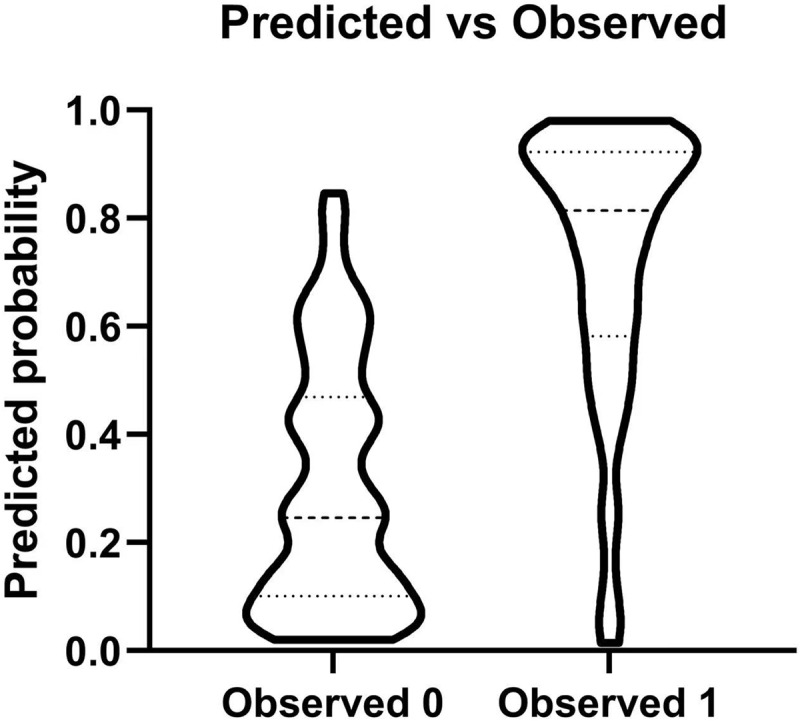
Predicted probability of multiple logistic regression. Legends: The positive predictive value of the model was 79.32%, and the negative predictive value was 79.35%.

**Figure 3.**
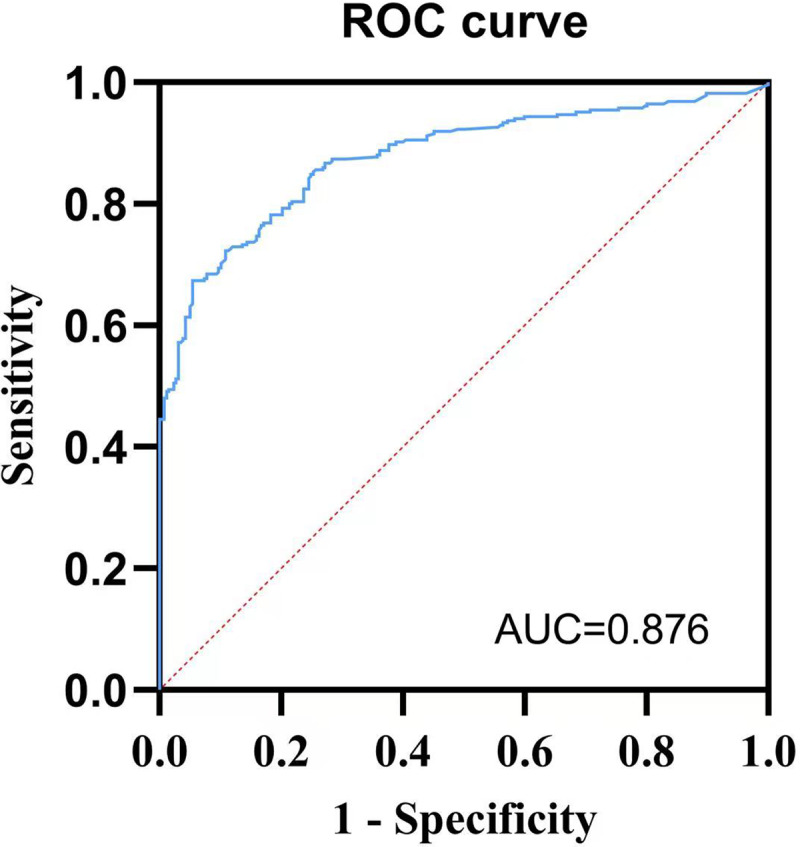
ROC curve of multiple logistic regression. Legends: Sensitivity refers to the proportion of true-positive results, and 1-specificity refers to the proportion of false-positive results. ROC curve showed the threshold value of the best prediction by the highest Youden index was 4.758, with a sensitivity, specificity, and Youden Index of 0.831, 0.862, and 0.693, respectively, and the AUC was 0.876.

The threshold value of the best prediction by the highest YI was 4.758, with a sensitivity, specificity, and YI of 0.831, 0.862, and 0.693, respectively. The positive predictive value was 79.32%, and the negative predictive value was 79.35% (Fig. 2). The ROC curve was shown in Fig. 3, with an AUC of 0.876. The above-mentioned parameters indicated a promising predictive value of the model.

## Discussion

The prognosis of EC in rural areas of China may be improved by increasing the consciousness and knowledge of EC prevention and treatment among GPs in rural areas. Therefore, it is of vital importance to understand GPs’ current knowledge proficiency of EC prevention and treatment in rural areas and analyze relevant influencing factors. To our knowledge, this study is the first research to investigate the above-mentioned topic. The study results indicated that knowledge proficiency of rural GPs of EC prevention and control still await to be improved. Sex, educational level, and comprehension of clinical practice guidelines for EC were significant predictors for their proficiency.

GPs play an important role in the health care system, and knowledge gaps in the professional field, such as epidemiology, early symptom identification, and screening methods of EC, may lead to unnecessary examination and overtreatment, or even delayed treatment. Our study showed poor knowledge proficiency of rural GPs in the field of EC prevention and treatment, especially in the aspects of risk factor identification and disease surveillance and follow-up. Accurate understanding of the risk factors for EC may facilitate the introduction of effective preventive measures as well as medical resource distribution, which are the fundamentals to reduce the incidence of EC (Li S *et al.*, 2021). Risk factors for EC vary with histological characteristics. It has been reported that esophageal squamous cell carcinoma is associated with smoking, drinking, cardiac damages, nutritional deficiency, etc., while esophageal adenocarcinoma is believed to be associated with high body mass index, smoking, reflux diseases, and a diet lacking in fruits and vegetables (Arnold *et al.*, 2020; Lander *et al.*, 2023). Therefore, correct identification of these risk factors may assist GPs to carry out public health propaganda, so as to decrease the incidence of EC. In addition, the follow-up visit is also an important aspect in the treatment of malignant tumors. Therefore, it is necessary for the GPs to master the key points of follow-up for EC. Our findings suggested that a series of policy interventions should be adopted to improve GPs’ knowledge proficiency of EC prevention and treatment.

The logistic regression analysis showed that sex, educational level, and comprehension of clinical practice guidelines for EC were significant predictors for GPs’ knowledge proficiency of EC prevention and treatment in rural areas. More specifically, female practitioners presented better knowledge than the male, which is in line with the conclusions of Li Y (Li Y *et al.*, 2021). The underlying reason for this phenomenon is still not clear. It is speculated that female practitioners tend to be more patient than the male (Veness *et al.*, 2019). In addition, the results indicated that as the educational level increase, GPs have better knowledge of EC prevention and treatment, suggesting that previous educational background may affect knowledge proficiency, which was also confirmed by previous studies (Hooja *et al.*, 2016; Viwattanakulvanid *et al.*, 2020). It is generally accepted that previous academic training may assist the GPs to formulate a mode of thinking that can help them in learning. However, due to the unequal distribution of health medical care personnel between urban and rural areas, rural medical care personnel are always insufficient in number, education, and professional skills (Li *et al.*, 2017). Therefore, it is necessary to formulate specific policy incentives for medical students to divert part of the talents to rural areas (Bao and Huang, 2021). Educators in medical colleges should enable medical students to understand the national medical reform policy and the work content of GPs during their study in colleges, so that general medicine teaching can start earlier and help medical students to think about their future career choices.

Medicine is a practical discipline, and accumulated clinical experience is an important source of knowledge (Sharon *et al.*, 2018). However, in our study, the knowledge of EC prevention and treatment did not increase along with advance in clinical practice experience (such as the extended years of work experience, professional title and outpatient visits), and even the knowledge level of those with working experience of more than 10 years showed a downward trend, which may be related to current rural medical practice. In rural areas, the job duties of GPs mainly include routine health examination, preventative care, vaccination, health promotion advice, follow-up, etc. (Qin *et al.*, 2023), which could be established and comprehended early in career (Rahimi-Ardabili *et al.*, 2021), while more advanced clinical knowledge were learned from books and training rather than practice. Practitioners with more years of working experience may be affected by knowledge forgetfulness, knowledge aging, and other factors, thereby the longer the working years, the lower the knowledge level.

Clinical Practice Guidelines (CPGs) are management protocols that cover recommendations on prevention, diagnosis, treatment, and long-term management to aid clinical decision making (Ravindra *et al.*, 2020), which are developed for the unification of clinical practice as well as safeguard mechanism that the medical services are provided on an evidence-based basis. Employment of CPGs in clinical practice is a core skillset to improve the quality of medical services (Albarqouni *et al.*, 2018). GPs generally acknowledge the importance of CPGs, while only a few actively use the database to learn CPGs, and more often they learn CPGs through continuing education (Young and Ward, 2001). Our study showed that only 15.5% (54/348) of GPs have studied the clinical practice guides of EC, and in-depth comprehension of CPGs could improve GPs’ knowledge proficiency of EC prevention and treatment in rural areas. Therefore, the government should strengthen the continuing education of GPs in rural areas and ensure that rural GPs have the opportunity to receive specialized CPG training courses.

This study had some limitations. First, this study was a cross-sectional study, with the result sample mainly composed of males. This limits the generality of the data to the wider population. Second, causal inferences could not be made due to the cross-sectional design of the study. Furthermore, our questionnaire did not provide a more detailed classification of the sources of GPs. A horizontal comparison of the knowledge proficiency of GPs from different rural areas cannot be performed. In future research, we will expand the sample size and refine the questionnaire content for more in-depth analysis

## Conclusions

The study results indicated that knowledge proficiency of rural GPs of EC prevention and control still await to be improved. Sex, educational level, and comprehension of clinical practice guidelines for EC were significant predictors for their proficiency.

## Supporting information

Zhang et al. supplementary material Zhang et al. supplementary material

## Supplementary material

For supplementary material accompanying this paper visit https://doi.org/10.1017/S1463423623000701
