# Supplementary material for: Influencing factors of knowledge proficiency of general practitioners in rural China for esophageal cancer prevention and treatment: a cross-sectional study
Source: Prim Health Care Res Dev. 2024 Feb 12;25:e9. doi: 10.1017/S1463423623000701 (PMC10894722; doi:10.1017/S1463423623000701)
Supplement: Zhang et al. supplementary material [file S1463423623000701sup001.doc]

Table S1. The self-designed questionnaire of GPs' knowledge in EC prevention and treatment

|  | Items |
| --- | --- |
| Risk factor identification | 1.Do you think long-term smoking will increase the chance of developing esophageal cancer? |
|  | 2.Do you think drinking a lot of alcohol will increase the chance of developing esophageal cancer? |
|  | 3.Do you think a low-salt diet will increase the chance of developing esophageal cancer? |
|  | 4. Do you think the habit of eating hot food will increase the chance of esophageal cancer? |
|  | 5. Do you think eating too fast will increase the chance of esophageal cancer? |
|  | 6. Do you think abdominal obesity will increase the chance of esophageal cancer? |
| Screening and diagnosis | 7. What do you think is the starting age for esophageal cancer screening in China? |
|  | 8. What do you think are the symptoms of people with early esophageal cancer? |
|  | 9.What do you think are the symptoms of people with advanced esophageal cancer? |
|  | 10.Which of the following diseases do you think may develop into esophageal cancer? |
|  | 11. Which of the following do you think is the best method for diagnosing esophageal cancer? |
|  | 12.What do you think are the commonly used serum tumor markers for esophageal cancer? |
| Treatment method for different stages | 13. Which of the following methods do you think can treat early esophageal cancer? |
|  | 14. Do you think early esophageal cancer can be completely cured? |
|  | 15. What do you think are the treatment options for advanced esophageal cancer? |
|  | 16. Which of the following conditions is suitable for chemotherapy? |
| Disease surveillance and follow-up | 17. What is the follow-up time of gastroscopy after endoscopic treatment for early esophageal cancer? |
|  | 18. What is the follow-up time of gastroscopy for precancerous lesions? |
|  | 19. What are the main follow-up contents for patients with esophageal cancer after surgery? |
|  | 20. What are the main follow-up contents for patients with esophageal cancer after radiotherapy and chemotherapy? |
